# Dr. Baillarger on the Pellagra

**Published:** 1848-07-01

**Authors:** 

**Affiliations:** Physician to the Asylum of the Salpêtrière


### THE PELLAGRA.

(*Address delivered at the Annual Meeting of the Académie Royale de Médecine, by* Dr. Baillarger, *Physician to the Asylum of the Salpêtrière. Communicated by* Dr. Sigmond.)

The pellagra, a disease already the subject of study for more than a century by a number of observers, is now generally known. Its seat is ascertained to be Lombardy, in some parts of Spain, and the South of France; it rages almost entirely in the country, and principally amongst the poorest peasants; it is also remarked, that although at its first attack it is very trifling, yet it is not generally long before the most serious symptoms are developed. To an erythema of the hands, which sometimes scarcely attracts the attention of the invalid, quickly succeed obstinate diarrhœa, gradually producing extreme emaciation; infiltration takes place into the limbs, serous effusions into the cavities, and then, after many years of suffering, the patient sinks under extreme marasmus. Amongst the symptoms remarked by authors are, meningitis, quickly fatal; suicidal melancholy, and mania. The variety of forms under which the disease appears, explains the numerous appellations which have from time to time been given to it. Amongst these denominations is one which indicates a very frequent order of symptoms, which exhibit themselves in their most aggravated form,—the paralysis scorbutica of Louis Aldalli. This author is not so much struck with the cutaneous erythema, nor the gastro-enteritis, nor the delirium, which characterize the disease, as with the paralysis and the scorbutus. If it were desirable briefly to sum up the symptoms of this species of paralysis, it would be by stating that it affects all the limbs at the same moment, but that its progress is slow and progressive, that it is accompanied by disorder of the mind, which terminates in insanity. It is impossible, whilst reflecting upon this abolition of mind and of muscular power, not to recal to mind that there exists in our lunatic asylums a disease, unfortunately too frequent, which consists also of abolition of mental and of muscular power. Struck with this resemblance, I attempted, at the meeting of the 3rd of August last, to demonstrate the striking analogy which appears to me to exist between the paralysis of those who labour under the pellagra, and that of the insane. I restricted myself to speaking of the analogy. In fact, there was wanting in the descriptions upon which I relied several important symptoms, whose absence could not permit me to regard the two diseases as identical. These symptoms are, especially, hesitation of speech, and the delusion which is manifested by ambitious hallucinations. Stuttering is one of the most constant symptoms of the paralysis of the insane, and it is decidedly one of the most valuable diagnostic signs that we possess of the first onset of the disease. As for the ambitious hallucinations, its absence would at once establish a marked difference between the diseases.

The singular fact, that the monomania of grandeur is always accompanied by general paralysis, however inexplicable it may be to psychologists, is, in fact, one of the best demonstrated facts in pathology. It is necessary to bear in mind, for the complete elucidation of the observations which follow, that this delirium may exist in different degrees of intensity. Amongst the paralytics of whom M. Calmeil speaks, some are in possession of provinces, some of empires, some of worlds, but there are others whose fancy is infinitely more limited. Amongst some even, there is nothing very observable beyond a sense of extreme self-satisfaction, of confidence in their own strength, and in the duration of their lives. These varieties, it must be remembered, do not change the leading principle, a circumstance so remarkable and so common, that it was pointed out by Hallam at a time when this general paralysis was actually unknown in France. "These insane persons," observed he, "whilst they cannot support their limbs, consider themselves full of vigour, and capable of the greatest efforts. Whatever pity," he adds, "such a state is calculated to inspire, it is nevertheless fortunate for these afflicted people that their confidence and their pretensions are exaggerated in the same ratio as their degradation is real. The sight of these people, almost incapable of movement, deprived entirely of the power of moving, stuttering with great difficulty a few unintelligible words, often covered with sores, yet still preserving in this miserable state the most brilliant illusions, is a contrast too striking not to have made a vivid impression upon observers."

But if the symptoms of paralysis pellagrosa are far from being identical with those of the paralysis of the insane, the opinion of authors upon the seat of the two maladies tends still further to separate them. The paralysis of pellagra, as the lesions observed in patients have demonstrated, is to be referred to the spinal marrow, whilst the general paralysis of our asylums is, on the other hand, more generally ascribed to a purely cerebral affection. With the exception of certain cases, the spinal marrow has been uniformly found healthy. Such, then, are the principal differences which appear to exist between these two states, in which paralysis is present. These differences—are they real? Ought these two affections to be considered entirely independent? or, on the other hand, is the paralysis of those afflicted with pellagra incompletely studied, at least, in the greater number of instances, and therefore not so well known as the malady so admirably described by Bayle and Calmeil? These questions have appeared to me to be worthy of being examined with care, as much that we might become intimately acquainted with the history of pellagra as well as that of general paralysis. This inquiry required new researches, and it is the researches made in different hospitals of Lombardy that I wish to have the honour of making known to the Academy. I should fear to trespass on your time if I were to relate the twelve cases which form the basis of my labours. I shall therefore confine myself to the detail of the first.

A peasant, named Garaviglia, forty-six years of age, entered the great hospital of Milan, afflicted with pellagra, on the 21st of June last, for the purpose of taking baths. He soon gave signs of madness, and was admitted into the receptacle of the insane. Three months afterwards he was transferred to the chronic ward. This transferring from the larger ward to the chronic cannot take place without a consultation of three physicians. This is the rule of the hospital. In this consultation a few words described the nature of the disease. Marasme pellagreux, tabes pellagrosa, was the diagnostic placed upon Garaviglia's ticket, and his insanity was ascribed to a pellagra some time existing. When I examined Garaviglia, he offered all the symptoms of general paralysis in an extreme degree; his physiognomy exhibited stupidity; his memory was altogether gone. He had an extreme embarrassment in speaking; trembling and feebleness of limbs, especially in the upper extremities; the legs were drawn back; standing was quite out of the question, and he had a large eschar upon the sacrum.

This man did not appear to have any ideas of grandeur, but upon his arrival at the hospital he had made himself remarkable by his magnificent promises to the people around him, by the complacency with which he spoke of his herds of cattle, of the number of his horses, &c. He had, besides this, two other symptoms which appeared to me worthy especially of notice. The first was a grinding of the teeth, a very frequent symptom amongst paralytics in our asylums, and which Strambio has pointed out as a sign of approaching death. It is particularly remarkable, and the sound is produced by the rubbing of the large molares against each other. The second symptom of which I would speak, is a movement, almost automatic, of the lips and jaws, and which has been compared by the author of whom I have just spoken, to the action of tasting. *Quidam oris motus quasi hominis quid sapidum gustantis.* This last symptom had not been observed amongst the paralytics in our institutions, but since my return I have ascertained that it is not peculiar to paralysis pellagrica. My colleague, M. Trelat, has already observed it in a paralytic under his care, and I now see it clearly in a case under my own management. The second observation is that founded upon the case of a peasant, aged forty-five, sent from Monza to the hospital of Senavra, at Milan. It was a female suffering under pellagra. She had all the symptoms of general paralysis, characterized by embarrassment of speech and ideas of ambition. I cite this, because the nature of the delirium is clearly indicated in the certificate of the physician of the Canton of Monza, Dr. Mezotti. It appears from this certificate, that for three successive years she had in the spring, besides the erythema pellagrosa, a monomania of prodigality. She laid claim to the possession of a large fortune, and thought only of living in great style, and ordered magnificent dinners at the hotels.

The third fact is one of those which I collected at Brescia. It is remarkable, from the excess of the ambition displayed during the delirium. The invalid, named Martinelli, was a fisherman at Iseo. The pellagra had existed many years. When I saw him, he stammered, and could scarcely sustain the weight of his limbs, but he still retained all the marks of great cerebral excitement. He constantly repeated that he was the Emperor Napoleon, and spoke of nothing but his immense treasures. This poor invalid seemed, above all, to insist upon his having a claim to be the proprietor of the Lake of Iseo. He wished to change the name of the lake, and was desirous that it should be called Lake Martinelli. In one of the cases of general paralysis which I observed, the pellagra was preceded by that which is called in Lombardy the "master's malady," a sort of hypochondriasis which is very common amongst the peasants. This appearance of cutaneous erythema occurs very frequently; so much so, that some author has gone so far as to assert that the pellagra and this species of hypochondriasis are one and the same malady. Strambio has had no difficulty in refuting this opinion, without, however, being able to deny that the two diseases have a singular association. This point seems to me to be of essential importance in the etiology of pellagra. I will add, that it is not only hypochondriasis, but almost all the neuroses which constitute a decided predisposition to this disease. It is even very rare to see a development of erythema upon the hands in the lunatic asylums in those who have never had any signs of pellagra before they entered the hospital. Excess of drink and delirium tremens also predispose to erythema pellagrosa. This fact has been placed beyond doubt, by the researches of Dr. Nobili, and by those which have been recently made by the commission at Piedmont. But it is by the hereditary disposition that the link between pellagra and madness may be rendered more easily intelligible. Many persons afflicted with pellagra spring from insane parents, and many insane persons from pellagrose parents. The development of one or other disease is dependent upon occasional causes. I will only cite here the following fact, which belongs to the history of general paralysis.

A peasant of the neighbourhood of Bergamo had three children, two sons and a daughter. The eldest son and the daughter had, like the father, the pellagra. The second son left the labours of the field, and became a servant in the town. He thus escaped the erythema pellagrosa, but was soon attacked by ambitious madness, and became paralytic. Besides this connexion between pellagra and madness, there is a fact which I hope I shall be excused for now mentioning. In the session of 1844, I had the honour to read before this Academy a memoir upon hereditary madness, (published in the ninth volume of the Bulletins of the Academy.) My object was to prove that hereditary madness is more generally transmitted from the mother than from the father, and more generally from the mother to the females, and from the father to the boys. At the commencement of the same month, M. Calderini, who could have no knowledge of my labours, published upon hereditary pellagra statistic conclusions which exactly coincided with my own observations. This, which is at least a coincidence, ought to be mentioned. I will add that the principal cause of the propagation of pellagra is hereditary predisposition. This fact, well studied and well understood, ought, in my opinion, to overthrow the hypothesis, otherwise well supported, of the exclusive action of maize. How can it be admitted that a single toxocological agent could bring into action the hereditary germ; how can it be understood that by a singular exception the symptoms of poisoning produced by maize could be transmitted from parent to child? The hospitals of Venice contain many pellagrose patients, and I met there a certain number of paralytics. One among them, at the outset of the disease, was a prey to suicidal ideas. This symptom, so common, as is known, in pellagra, has been differently explained. I will try to demonstrate that, most probably, one of the principal causes has been neglected.

It has been asserted that pellagrose patients destroy themselves, to put an end to the pains they have to endure; it is said, also, that the despair which seizes upon these unfortunate persons takes it origin from the conviction of the incurableness of the disease. This explanation might be admitted, if the suicidal tendency was observed amongst the pellagrose who are not insane; but this is not the case. The invalids who have sound reason do not seek, or very rarely seek, to destroy themselves. A young physician attached to the hospital at Brescia declares, in an admirable work upon Pellagra, that he has never seen the tendency to suicide, and above all, adds he, to the celebrated hydromania of Strambio. M. Calderini, who has marked with minute care the symptoms which presented themselves in a thousand pellagrose patients of sound mind, never speaks of the idea nor of attempts at suicide. This symptom, then, belongs almost exclusively to pellagrose madness, and its extreme frequency seems to be explained in the following manner:—

Ideas of suicide are never observed to be more frequent than in that species of insanity to which Esquirol has applied the term, *demence aigüe;* and Georget, *stupidité.* They exist in at least a third part of those who are thus diseased. It results from this observation, that to give the proportionate number of suicides amongst a given number of insane, it ought to be ascertained beforehand whether the *demence aigüe* has been of frequent or of rare occurrence. The pellagrous insanity offers a singular exception. In our asylums we scarcely find three cases in a hundred which are *demence aigüe;* on the contrary, in the pellagrous insanity, according to the researches of M. Pelt, physician to the insane at Venice, the proportion is thirty in a hundred. The *demence aigüe* is, then, ten times more frequent amongst pellagrous insane than in our asylums. Is it not, then, evident that there is here one of the principal causes of the numerous cases of suicide remarked in pellagra? Other facts also come in support of this explanation. "The pellagrous," says Strambio, "destroy themselves without giving any sign of passion, or menacing any one." It is precisely thus when suicide occurs in *demence aigüe.* These lunatics, motionless, inert, silent, and apparently stupid, seek self-destruction without giving the slightest sign of excitement; one might say the act was purely automatic. It appears to me, then, that this great proportion of suicides amongst the pellagrous insane may, in a great part at least, be explained by the number of cases of *demence aigüe.* This last fact is otherwise easily to be understood. Stupidity is most generally to be noticed in subjects of debilitated constitution; it is to be seen after losses of blood, after long abstinence. Sydenham has described it as occurring after intermittent fever of long standing. It is, therefore, that the pellagrous, arrived at the second or third stage of the disease, are often attacked with this species of madness. I return to pellagrous paralysis. Besides the observations which I have collected, I have thought it right to consult the registers of the hospitals; and those of the Senavra of Milan have furnished me with several interesting facts; amongst these, there is one in which the embarrassment of pronunciation amongst the paralytics is described in a manner as striking as true. "The words of the diseased," says the author of the remark, "were *troncati con solfeggio.*" Nothing could be more exact. The pronunciation of paralytics produces to a certain degree a sort of solfeggio. Thus, when M. Calmeil wished to describe this pronunciation, he found no better means than to separate each syllable by a line, exactly as the notes of music are separated from each other. Besides this fact, there are some curious remarks upon what the author calls pellagrous encephalitis. This disease, says he, is more frequent than is imagined. It commences by an inflammation of the membranes, which gradually extends to the brain. Upon morbid examination, the membranes are found thickened, infected, infiltrated with plaster lymph, the cortical substance very red, and the brain itself more or less softened throughout its substance. All this bears a resemblance to that which is observed in the paralysis of the insane, and there are in both instances the same anatomical changes. Pellagrous paralysis, in the second stage, may easily be confounded with another state very different: we know that the pellagrous have often a tottering walk, a kind of *titubation* very poetically described by Strambio, sometimes there are cases in which this is easily cured; on the contrary, there are others totally incurable. The first have a tottering which may almost be called convulsive; the second, a simple feebleness of limbs, especially the lower, sometimes accompanied by trembling. The convulsive titubation appears to me to be to paralysis that which *la demence aigüe* is to madness. I will add, that these general symptoms allow very often a distinction, of which the importance may be imagined in the forming a prognosis. It remains for me to point out the result of the autopsies which I made in Lombardy, to ascertain if pellagrous paralysis is a cerebro-spinal or a purely cerebral affection, and to demonstrate, under the head of anatomical alterations, the convulsive titubation which is not paralytic; but the examination of these questions will lead me on too long, and I find myself compelled to postpone these subjects till I can give the result of my extended labours upon pellagra. These labours only can confirm the observations made upon those symptoms which have already been described, and which teach us the identity of pellagrous paralysis with insanity. Before concluding, I think it right to remark, that inquiry into paralysis pellagrosa completely confirms the union between the monomania of grandeur and general paralysis. I have encountered in Lombardy that special monomania amongst many paralytics not affected with pellagra, and I can assert that such cases are not so uncommon as has been said in the hospitals of Milan. One of those I saw pretended to be the prophet Elias; one would have imagined that he had only come into the hospital to relieve himself from the inconvenience of receiving the homage of the multitude out of doors. A poor artist, named Ambrose, would do nothing but regenerate the world; his ambitious dream, described in all its details in the asylum of Senavra, is only an exaggerated painting of the golden age. In another month, all disease would disappear from the earth, and death cease to strike. The men would everlastingly be thirty-five, and the women twenty-five years of age; life would only be one long-continued fête.

As may be seen, the pretensions of the paralytic in Lombardy do not yield to those of the deranged in our asylums. This singular union of the ambitious monomania with paralysis is the same everywhere. In vain has it been attempted to explain this by some of the ideas which prevail in society. That which shows the little value of such explanation is, that this alliance between paralysis and ambition was pointed out as well in the last as in the present century, that it is found amongst peasants afflicted with pellagra, careless of the future generally, as well as amongst individuals engaged in the pursuit of wealth and of honour.

It remains, then, only to point out this as a fact which will remain one of the most curious in the history of the disorders of the mind.

---

